# “I know that exercise is very good for me”: perception of pediatric patients with cystic fibrosis about physical exercise program

**DOI:** 10.1590/1984-0462/2026/44/2025358

**Published:** 2026-08-24

**Authors:** Cristiane Cenachi Coelho, Marcella Guimarães Assis, Evanirso da Silva Aquino, Lídia Cunha de Oliveira, Priscila Souza, Anny Caroline Silva Ribeiro Oliveira, Fernanda Cordoba Lanza, Danielle Aparecida Gomes Pereira

**Affiliations:** a Universidade Federal de Minas Gerais, Physiotherapy Department, Postgraduate Program in Rehabilitation Sciences, Belo Horizonte, MG, Brazil.; b Fundação Hospitalar do Estado de Minas Gerais, Hospital Infantil João Paulo II, Rare Diseases Unit, Belo Horizonte, MG, Brazil.; c Universidade Federal de Minas Gerais, Department of Physiotherapy, Belo Horizonte, MG, Brazil.

**Keywords:** Exercise, Cystic fibrosis, Behavior, Self-management, Exercício, Fibrose cística, Comportamento, Automanejo

## Abstract

**Objective:**

To explore and understand the perception of children and adolescents with cystic fibrosis diagnosis regarding the integration of a home physical exercise program into their daily routine.

**Methods:**

This study used a qualitative method. Children and adolescents diagnosed with cystic fibrosis who were being followed up in a home exercise program participated by answering a semi-structured, face-to-face interview. The data were analyzed using the thematic analysis technique.

**Results:**

Data from 38 participants were collected: 29 were pre-school/school ages (between six and 12 years old), nine were adolescents (between 13 and 16 years old). The median age of the participants was ten (interquartile range 8–10) years. Three thematic categories were identified from analyzing the interviews using a qualitative, descriptive and exploratory methodology: 1) I feel that I am better; 2) I did almost everything (all the exercises of the daily routine); 3) “If I could remove one exercise…” (the expression of preferences).

**Conclusions:**

The perception of children and adolescents with cystic fibrosis diagnosis who completed the home exercise program was that it improved their health condition. This type of program positively changed the participants’ perception of their symptoms and may allow participants diagnosed with cystic fibrosis to have greater engagement and adherence to their care.

## RESUMO

**Objetivo:**

Explorar e compreender a percepção de crianças e adolescentes diagnosticados com fibrose cística sobre a integração de um programa de exercícios físicos domiciliares à sua rotina diária.

**Métodos:**

Este estudo adotou um método qualitativo. Crianças e adolescentes com diagnóstico de fibrose cística, acompanhados em um programa de exercícios domiciliares, submeteram-se a uma entrevista semiestruturada presencial. Os dados foram analisados por meio da técnica de análise temática.

**Resultados:**

Foram coletados dados de 38 participantes, sendo 29 em idade pré-escolar/escolar (entre seis e 12 anos), e nove adolescentes (entre 13 e 16 anos). A mediana de idade dos participantes foi de dez anos (intervalo interquartil — IIQ 8–10). Três categorias temáticas foram identificadas com base na análise das entrevistas, utilizando uma metodologia qualitativa, descritiva e exploratória: 1) “Eu sinto que estou melhor”; 2) “Eu fiz quase tudo” (o exercício na rotina diária); 3) “Se eu pudesse tirar um exercício…” (a expressão de preferência).

**Conclusões:**

A percepção das crianças e adolescentes que concluíram o programa foi de que houve melhora em sua condição de saúde. Este modelo de intervenção alterou positivamente a percepção dos participantes sobre os sintomas, além de promover maior engajamento e continuidade no autocuidado.

## INTRODUCTION

Cystic fibrosis is a multifactorial chronic condition with systemic dysfunction that destroys lung tissue, resulting in cardiovascular disorders, peripheral muscle impairment, reduction in contractile strength, increase in muscle fatigue, and impairment of physical capacity.^1-4^ Reduced functional capacity, particularly in children and adolescents, makes it difficult to perform activities such as playing.^5,6^ Thus, they often prefer isolation due to these restrictions.^7,8^

Treatment of cystic fibrosis aims to reduce symptoms, prevent complications, and improve functional capacity and well-being.^9^ Physical activity and exercise programs help in the maintenance of satisfactory levels of cardiorespiratory conditioning, strength, and physical fitness, which improve daily living activities and recreation.^5,10-12^ Considering the importance of exercise in childhood, to increase adherence and facilitate access for children and adolescents with cystic fibrosis diagnosis to exercise, home-based programs are promising strategies with proven efficiency.^13^

The knowledge, attitudes, and behaviors of patients with cystic fibrosis about their condition have already been investigated.^7,14^ However, studies addressing the auto-perception of children and adolescents about their general behavior with the inclusion of physical exercise into their daily lives are incipient. It is essential to listen to these patients to implement more effective programs, as this group tends to exhibit higher levels of demotivation, thereby reducing engagement in physical activity. Regarding this concern, conducting a qualitative study allows for an understanding of how patients perceive their disease, exploring feelings, experiences, and personal challenges that quantitative methods may not capture. This information helps guide interventions and strategies better suited to this group’s needs. Thus, this study aimed to explore and understand the perception of children and adolescents with cystic fibrosis regarding the integration of a home exercise program into their daily routine.

## METHOD

This is a qualitative study. The research was approved by the Research Ethics Committee of the Universidade Federal de Minas Gerais — number of the Certificate of Presentation for Ethical Appreciation (CAAE) 78556017.6.0000.5149 —, and the results were reported in accordance with the Consolidated Criteria for Reporting Qualitative Studies (COREQ). Participants and their parents/guardians read and signed the Informed Consent Form for the study.

The inclusion criteria were children and adolescents who were clinically stable and had two genetic mutations of the disease, aged 6–16. Participants were selected from a group of patients with cystic fibrosis at the Cystic Fibrosis Reference Center for Rare Diseases of the Hospital Infantil João Paulo II (a public philanthropic center). All participants who had completed a home-based aerobic exercise program were randomly selected from a research study on peripheral muscle tissue oxygenation and functional capacity. Recruitment and data collection were discontinued once thematic saturation was achieved.^15^ This point was determined through continuous analysis of the transcripts, as the research team observed that subsequent interviews did not yield novel insights or additional sub-themes regarding participants’ experiences with the physical exercise program. The consistency of the responses confirmed that the data set was sufficient to address the research question, as no further relevant information or themes emerged.

The home exercise proposed by the physiotherapy team included daily activities, lasting 30 minutes (at least 150 minutes per week),^12,16^ over six months. The aerobic exercises were simple to perform and tailored to participants’ characteristics, such as age, motor skills, and preferred activities (e.g., jumping rope, swimming, and interactive games). The resources available at home for family activities were also evaluated to inform activity recommendations. Participants and their families received a manual with the physical exercises and instructions for performing them. To monitor exercise intensity, a Borg scale from 0 to 10 was used. Everyone was instructed to maintain an effort level of five during the activities, which corresponds to an intense effort. At the beginning of the program, each participant received an individual calendar to record exercise frequency and how the activity should be performed, and to record a subjective perceived exertion scale (Borg scale) to monitor intensity during physical exercise. The parents/guardians of the children and adolescents were responsible for helping them with home activities and agreed to maintain telephone contact with the physiotherapy team of four physiotherapists.

Participants received messages on their mobile phones every two weeks, emphasizing the need to perform the exercises. In the first month of the program, the physiotherapy team made calls to check whether participants had any questions and whether adjustments were needed to any of the prescribed exercises. Every three months, participants undergo reassessments, and two follow-ups were carried out.

Data collection was performed using semi-structured interviews at the end of the physical exercise program, on the last day of follow-up appointments of children and adolescents, at the Cystic Fibrosis Reference Center for Rare Diseases of Hospital Infantil João Paulo II. The interview was developed based on expert consensus — three researchers: one with specific training in pulmonary rehabilitation, one senior researcher with expertise in qualitative studies, and the coordinating researcher for the study. This interview consisted of questions related to children’s and adolescents’ perceptions and experiences of carrying out a home exercise program, characterized by reduced symptoms and increased engagement in daily activities, as well as the positive and negative aspects of its execution. The interview also focused on issues related to the participants’ strengths/weaknesses in completing the exercise program (frequency) and suggestions for improvement in subsequent phases. A pilot study was conducted to assess whether the questions were understandable and/or adjustments were needed, and no changes to the interview guide were needed.

Interviews were conducted in a quiet, uninterrupted setting with the participant’s guardian present. The questions were administered face-to-face, in the same order for all participants, and responses were recorded according to their own narrative. No minimum or maximum time was set for the interviews. The interviews were recorded and later transcribed in full. All interviews were carried out by a single researcher. Identification of the children and adolescents was carried out by interview ID, sex, and age to guarantee anonymity. The genetic mutations of the disease and lung function variables were collected from the participant’s board. Lung function was considered normal when forced vital capacity (FVC) and forced expiratory volume in the first second (FEV_1_) were > 80% of the predicted value, and the FEV_1_/FVC ratio was within the normal range. Obstruction was classified as moderate when FEV_1_ was between 50 and 79% of the predicted value, and severe when FEV_1_ was < 50% of the predicted value.

The data were analyzed using thematic analysis, as proposed by Braun and Clarke.^17^ This analysis is divided into six phases:

1. familiarization with the data;
2. generation of codes;
3. searching themes;
4. reviewing themes;
5. defining and naming themes; and
6. producing the report^17^.

This type of analysis is a recursive process across the phases, involving continuous back-and-forth reading and interpretation of the data. To increase the trustworthiness of this qualitative study, the first author transcribed all interviews. Two researchers (authors 1 and 2) coded the transcripts independently and then collaboratively discussed and refined the themes before sharing the results with the last author. The research group held four meetings to discuss the themes and the best excerpts to exemplify them.

## RESULTS

Of the 60 children and adolescents who completed a home exercise program, 38 were included in this qualitative study due to data saturation. Of the 38 participants in this study, 29 were pre-school/school ages (between six and 12 years), and nine were adolescents (between 13 and 16 years old). The participants had a median age of ten (8–10) years, and 38% had normal lung function (Table 1). Clinical characteristics, including age, sex, and clinical profile, were similar between the 22 excluded patients and the study participants.

**Table 1:** Characteristics of the study sample.

Variables	Total (n=38)
Age, years old*	10 (8–10)
BMI, kg/m^2^ *	18.4 (15.8–21.6)
Sex n (%)	
Boys	23 (60)
Girls	15 (40)
Normal lung function (%)	29
Moderate lung obstruction (%)	25
Severe lung obstruction (%)	8

*Median age and the interquartile range.

BMI: body mass index.

**Figure 1:**
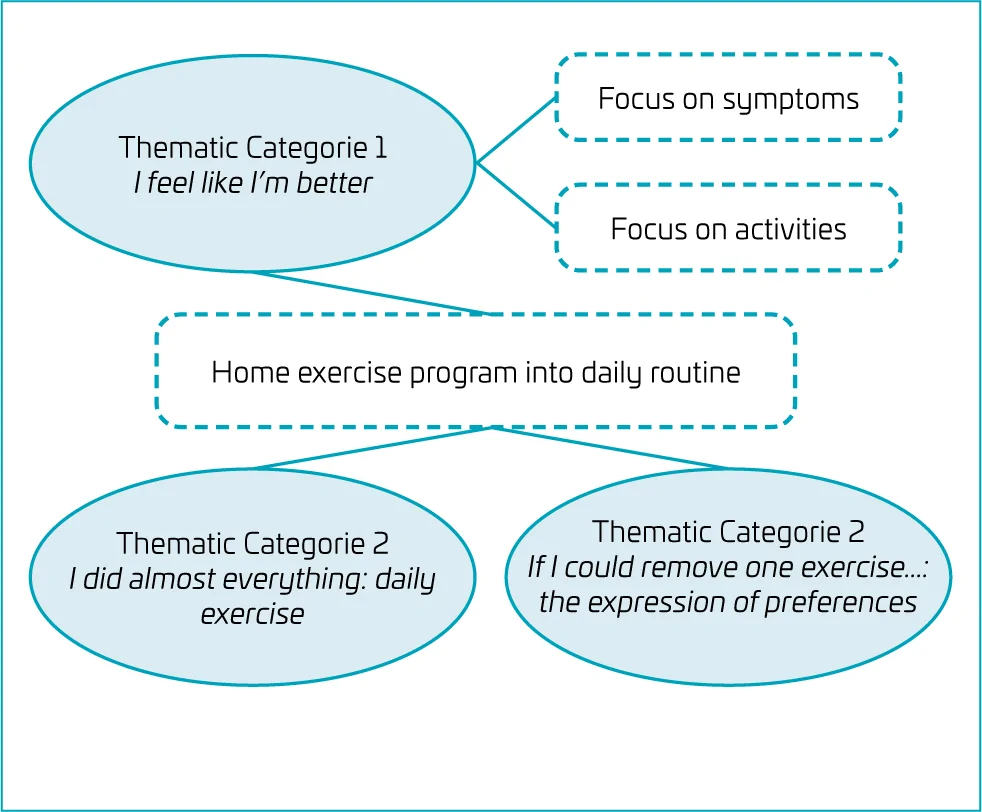
Organizational chart of the thematic categories.

Source: Author’s development based on thematic categories observed at patticipants’ answers.

After detailed data analysis, three thematic categories and two subcategories were organized, as shown in Figure 1.

Thematic category 1: *I feel like I’m better.* The interviewees reported improvements in their health status, characterized by reduced symptoms and increased engagement in daily activities.

Subcategory: *Focus on symptoms.* The participants reported that their inclusion in the exercise program resulted in better well-being and reduced pulmonary (cough, dyspnea, pulmonary secretion) and nutritional symptoms (weight loss and under-expected growth).

*“[...] Before doing the exercise program, I was very unwell, now I feel that I am better.”*
*(E4, female, 9 years old).*

*“[...] I’m not coughing a lot, just occasionally, I know the exercise has been very good for me. My mom also liked it. I gained more weight, I grew more [...].”*
*(E3, female, 9 years old)*

*“[...] I’ve gained almost a kilo since the last appointment. [...] I felt less tired. ’[...] I coughed less than usual when I felt the urge to cough.”*
*(E6, male, 9 years old).*

*“[...] My breathing improved. [...] Before I was hospitalized a lot, now I have only been hospitalized for decolonization every three months. I am not exacerbating the same as before. I also do not feel tired.”*
*(E36, male, 16 years old).*

Subcategory: *Focus on activities.* The interviewees reported improvements in carrying out daily activities at home and in physical exercise and sports at school:

*“[...] My breathing got better, which helped me organize myself at home in daily activities.”*
*(E23, female, 12 years old).*

*[...] At school, I kept hoping before that the teacher would choose me during physical education activities because she chooses only the good ones. Now, she also chooses me. [...] My friends and I have agreed to play dodgeball, football, shuttlecock, and flag-stealing, whereas before I could not do it.”*
*(E4, female, 9 years old).*

Thematic category 2: *I did almost everything* (daily exercise). Children and adolescents reported different frequencies of exercise at home, attributed to the need for rest, forgetfulness, and lack of time.

*“[...] I did almost everything. [...] I did not do it only on Saturdays and Sundays as these were my rest days.”*
*(E4, female, 9 years old).*

*“I didn’t do it for a few days (yesterday, the day before yesterday and Monday) because I forgot.”*
*(E6, male, 9 years old).*

*“[...] Sometimes my mother and I would both forget to do the exercises. Sometimes, we forget to write it in the diary.”*
*(E25, male, 15 years old).*

*“[...] I didn’t have time. [...] I exercised through the physical education class at school. [...]*
*(E1, male, 6 years old).*

One interviewee reported non-compliance with the exercise program due to the presence of symptoms such as cough.

*“[...] There were days when I was coughing a lot, so I couldn’t do everything.”*
*(E28, female, 14 years old).*

Thematic category 3: *If I could remove one exercise...* (the expression of preferences)*.* The children and adolescents gave their opinions about their required daily exercises. Group activities such as *queimada* (a Brazilian game like dodgeball), volleyball, and football were highlighted as preferred. On the other hand, they criticized and suggested removing the activities that required attention, coordination, and cardiorespiratory conditioning, such as jumping jacks and skipping rope.

*“[...] I like queimada, volleyball, and football.”*
*(E18, male, 12 years old).*

*“[...] I would like to do some kind of dance or ballet.”*
*(E24, female, 12 years old).*

*“[...] If I could remove an exercise, it would certainly be the jumping jacks since they are very difficult and tiring.”*
*(E4, female, 9 years old).*

## DISCUSSION

Children and adolescents with cystic fibrosis diagnosis in the present study recognized the relevance of exercise in improving their general health status. The efficiency of home-based rehabilitation has already been proven to improve quality of life in children and/or adolescents with cystic fibrosis,^13^ but poorly from the patient’s perspective.^18,19^

Based on the biopsychosocial model, understanding personal perspectives is relevant to promoting high-quality treatment.^18^ Additionally, understanding participants’ personal experiences is crucial for designing interventions that address physical, emotional, and social needs. In the present study, personal experience was considered, and participants reported that the home program reduced their symptoms, exacerbations, and fatigue, thereby improving their health status.

The home program, based on the interests of children and adolescents, allowed flexibility in the type of exercises prescribed by the physiotherapist. Participants’ characteristics, such as sex, age, motor skills, respiratory symptoms, socialization, preferred activities, and living environment, were important in determining the exercise program. This results in a better understanding by the participant and greater integration into the treatment.^8,20,21^ All these strategies contributed to the participants’ completion of the home daily exercise program, as they described. This confirms, once more, that exercise based on each patient’s personal perspective may improve motivation.

The parents/guardians showed an interest in monitoring and helping the participants choose activities to ensure they were in line with their routines. They were also responsible for receiving and passing on to their children the reminders about exercises sent via mobile phones by the physical therapy team. The responsible person’s confidence in the treatment’s effectiveness helps them monitor and organize it at home.^8,21,22^

Participants reported that they could perform activities at home and at school, which they previously could not. This, in turn, positively influenced their daily organization because they felt stronger and more energetic, and, according to the reports, they improved their participation in group activities. The independence in doing home activities and socialization can reduce the level of stress and the unwillingness of these children and adolescents to carry out the treatment.^23,24^

Regarding exercise routines in daily life, the participants often reported needing rest, experiencing forgetfulness, and lacking time. Although the exercises considered their preferences, we observed that they continued to have difficulty maintaining the frequency of exercise at home. Even with parental reinforcement, about 70% of children and adolescents still abstained from exercising on weekends. The diary, which recorded how and when the activities were performed, was intended to monitor and assist their routines. The lack of time reported by participants, particularly adolescents, may be associated with their independence and difficulty in establishing priorities for themselves. Other activities that do not involve exercise are usually prioritized.^25,26^ For this reason, to achieve favorable outcomes, it is important to establish training in partnership with their families to encourage physical activity.^8,22^ It is still important to highlight the inclusion of daily exercise in their routine to achieve the expected benefits.^24^

The improvement in self-perception following a physical exercise program can have lasting effects on how individuals relate to their own bodies and engage in physical activity. When participants recognize positive changes after exercise, or overall well-being, this heightened body awareness and confidence can foster greater motivation to maintain an active lifestyle. Over time, such experiences may strengthen the sense of self-efficacy, promote a more positive body image, and encourage long-term adherence to exercise, ultimately supporting both physical and psychosocial health.

There are some limitations of this study. First, we did not assess parents’ or guardians’ perceptions of their children’s exercise program. An approach directed at them could also have helped with the daily family routines involving their children’s exercise at home. The socioeconomic characterization can make a difference in the answers in a quality study. Unfortunately, we did not collect this data; however, all patients were recruited from a public center that primarily serves individuals with low socioeconomic status. Another limitation is lack of information on participants’ prior experience with physical activity and how the exercise program affected their body awareness. Furthermore, the broad age range of the participants (6–16 years) may introduce developmental biases, as younger children and adolescents possess distinct cognitive abilities and varying levels of autonomy in self-care. Nevertheless, the consistency of the themes across the sample suggests that the program’s benefits were perceived similarly across different ages. Finally, the potential influence of a phenomenon where individuals alter their behavior simply because they know they are being observed in a research or workplace setting (Hawthorne effect) cannot be entirely ruled out. However, the reported integration of exercises into the participants’ daily routines suggests a meaningful behavioral change that extends beyond the research environment.

In conclusion, the children and adolescents who completed the home exercise program reported improvements in health status because of their participation. This type of program can be a feasible strategy for tackling the disease, as it allows greater engagement and adherence to care and positively changes the perception of symptoms and functional capacity.

## Data Availability

The database that originated the article is available, with a corresponding author.
